# Magnetocaloric performance of the three-component Ho_1-x_Er_x_Ni_2_ (x = 0.25, 0.5, 0.75) Laves phases as composite refrigerants

**DOI:** 10.1038/s41598-022-16738-7

**Published:** 2022-07-19

**Authors:** Jacek Ćwik, Yurii Koshkid’ko, Konstantin Nenkov, Evgenia Tereshina-Chitrova, Małgorzata Małecka, Bruno Weise, Karolina Kowalska

**Affiliations:** 1 Institute of Low Temperature and Structure Research, PAS, Okólna 2, 50-422 Wrocław, Poland; 2 Leibniz IFW Dresden, Institute for Complex Materials, 01069 Dresden, Germany; 3 Institute of Physics CAS, Prague, 18221 Czech Republic; 4 Faculty of Chemistry, Wrocław University of Science and Technology, Norwida 4/6, 50-373 Wrocław, Poland

**Keywords:** Engineering, Materials science, Ferromagnetism, Magnetic properties and materials, Phase transitions and critical phenomena, Thermodynamics

## Abstract

To date, significant efforts have been put into searching for materials with advanced magnetocaloric properties which show promise as refrigerants and permit realization of efficient cooling. The present study, by an example of Ho_1−x_Er_x_Ni_2_, develops the concept of magnetocaloric efficiency in the rare-earth Laves-phase compounds. Based on the magneto-thermodynamic properties, their potentiality as components of magnetocaloric composites is illustrated. The determined regularities in the behaviour of the heat capacity, magnetic entropy change, and adiabatic temperature change of the system substantiate reaching high magnetocaloric potentials in a desired temperature range. For the Ho_1−x_Er_x_Ni_2_ solid solutions, we simulate optimal molar ratios and construct the composites used in magnetic refrigerators performing an Ericsson cycle at low temperatures. The tailored magnetocaloric characteristics are designed and efficient procedures for their manufacturing are developed. Our calculations based on the real empirical data are very promising and open avenue to further experimental studies. Systems showing large magnetocaloric effect (MCE) at low temperatures are of importance due to their potential utilization in refrigeration for gas liquefaction.

## Introduction

The magnetocaloric effect discovered by Weiss and Piccard^1^ in 1917, consists of heating or cooling of a magnetic material under the magnetic field variation. The nature of MCE was explained, and its practical use to reach ultralow temperatures via adiabatic demagnetization was suggested independently by Debye^2^ and by Giauque^3^. To date, it is still one of the most used techniques to reach very low temperatures. Magnetic refrigeration based on the magnetocaloric effect has become an attractive alternative to conventional cooling methods owing to its energy efficiency and ecological safety. Up to now, an intensive search for materials suitable for the use as the working body of magnetocaloric refrigerators is under way^4–9^. All magnetic materials intrinsically show MCE, although the intensity of the effect depends on the properties of each material. The phenomenon is due to the coupling of magnetic sublattice of a solid with an applied magnetic field, which changes the magnetic contribution to the entropy of the solid. In the case of conventional MCE, isothermal magnetization reduces the entropy of a magnetic material, which subsequently can be cooled by adiabatic demagnetization, like the gas compression. In a reversible process, demagnetizing restores zero-field magnetic entropy of a system. Generally, MCE is defined by the isothermal entropy change, Δ*S*, in an isothermal process and by the temperature change, Δ*T*_ad_, in an adiabatic process. The values of the above magnetocaloric potentials usually are highest in the vicinity of a magnetic ordering temperature and decrease smoothly to zero beyond the magnetic phase transition region. The other correlated parameters that allow one to determine the magnetocaloric performance of magnetic material are the refrigerant capacity (*RC*) and relative cooling power (*RCP*) or temperature averaged entropy change (*TEC*)^10^.

The search for new materials for cryogenics among RNi_2_ (R—is a rare-earth metal) compositions, has its base in their structural, magnetic, and thermodynamic properties. Magnetocaloric cooling with the rare-earth-based Laves-phase materials offers higher efficiency for liquifying gases compared to conventional methods, e.g., hydrogen which is of great interest as an energy carrier in the decarbonization of the economy.

The RNi_2_ compounds belong to the Laves phases and crystallize with the formation of a simple regular MgCu_2_-type structure (C15). However, the majority of the RNi_2_ compounds crystallize in a cubic structure characterized by regular arrangement of vacancies at the rare earth sites, which stabilize these compounds in a structure derived from the ideal C15 cubic structure^11–13^. The ordering of the R vacancies on special lattice sites leads either to a tetragonal^14^ or cubic superstructure^12,13,15^. The superstructure derived from C15 can be described within the space group *F*-43 m and is characterized by the doubled lattice parameter *a* compared to the C15 structure^16^. The R atoms occupy five different crystallographic sites, whereas the ordered vacancies are located only at one of these 5 sites, namely, the 4*a* sites^15^. However, the 4*a* sites are not completely empty, and the occupancy varies among the investigated RNi_2_ compounds. This variation is due to different sizes of different R atoms occupancy and can also depend on other factors, such as, e.g., the starting stoichiometry of samples.

The RNi_2_ compounds are characterized by high localized magnetic moments originating from the incompletely filled 4*f*-electron shell of lanthanides. The non-magnetic state of Ni atoms in these compounds is the cause of low magnetic ordering temperatures because the range of wave functions derived from lanthanides is lower than the interatomic distances, and 4*f*-4*f* interactions are weak. The majority of RNi_2_ compounds are found to be ferromagnetically ordered at low-temperatures and, upon ordering, exhibit the second-order magnetic phase transition^17,18^. These features of RNi_2_ compounds determine the marked MCE and, hence, their promise as cryogenic refrigeration materials.

To provide the effective operation in the ideal Ericsson magnetic regenerator cycle, a magnetic working material should have a magnetic entropy change − Δ*S*_mag_ that is constant in the cycled temperature span^19^. The above considerations determine the possibility to make a “table-like” temperature dependence − Δ*S*_mag_ (*T*), namely, to reach the almost unchanged significant value of − Δ*S*_mag_ over a desired temperature range. In particular, it can be done with the Ho_1−x_Er_x_Ni_2_ compositions, for which the Curie temperatures (*T*_C_) range between 13.5 and 6.5 K for HoNi_2_ and ErNi_2_^20^, respectively. It should be noted that the “table-like” behavior of − Δ*S*_mag_(*T*) is the essential requisite for an ideal Ericsson-like refrigeration cycle^21^.

The purpose of this work is to characterize the structure and magneto-thermodynamic properties of Ho_1−x_Er_x_Ni_2_ and to analyze their evolution in accordance with the substitutions in the rare-earth sublattice. We focus on the practical magnetocaloric aspect of the Ho_1−x_Er_x_Ni_2_ solid solutions. Direct and indirect measurements of magnetocaloric potentials in a wide magnetic-field range allow us to extend the knowledge on the magnetocaloric nature of considered compositions with the magnetic dilution determining their properties as composite refrigerant components.

## Results

### Structural analysis

**Figure 1:**
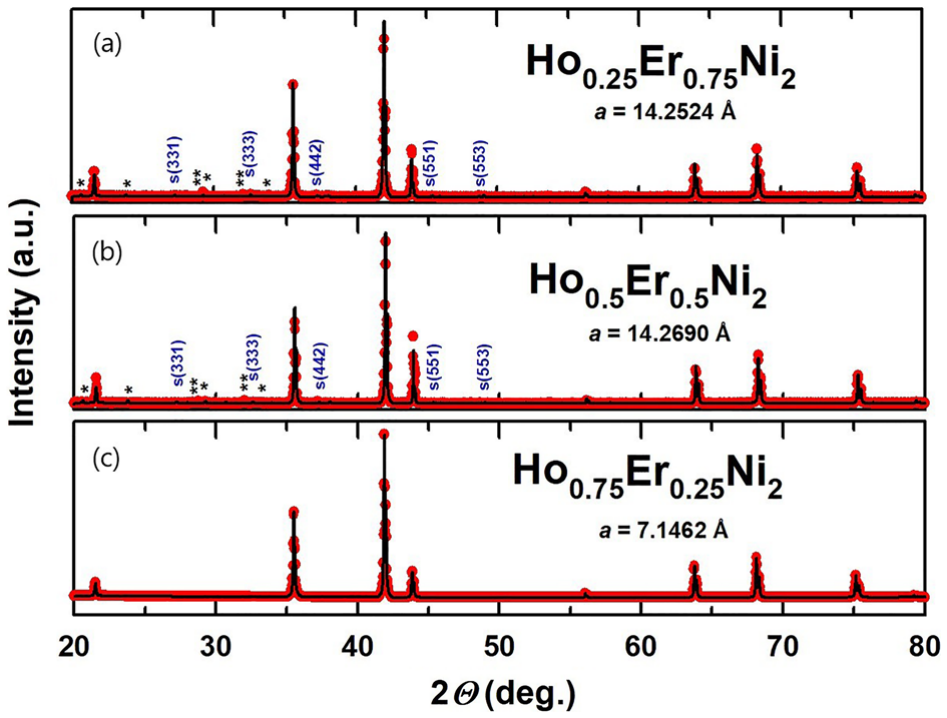
Results of the Rietveld refinement of the room-temperature XRD patterns taken for Ho_0.25_Er_0.75_Ni_2_ (**a**), Ho_0.5_Er_0.5_Ni_2_ (**b**) and Ho_0.75_Er_0.25_Ni_2_ (**c**). Peaks marked with S correspond to the superstructure (*F*-43 m space group) and peaks for the rare-earth (Ho,Er)_2_O_3_ oxide phases are marked with * and **.

The XRD patterns recorded for the Ho_1-x_Er_x_Ni_2_ solid solutions at room temperature were analyzed by the Rietveld method and are depicted in Fig. 1. Through the substitution of erbium for holmium in Ho_0.5_Er_0.5_Ni_2_ and Ho_0.25_Er_0.75_Ni_2_, the ordering of R vacancies preserved in the structure of HoNi_2_ phase takes place, and the 2*a* cubic superstructure (space group *F*-43 m) forms, indicated by indexed peaks marked with S in Fig. 1 a, b. For the Ho_0.75_Er_0.25_Ni_2_ stoichiometric composition, in contrast to Ho_0.5_Er_0.5_Ni_2_ and Ho_0.25_Er_0.75_Ni_2_, this effect is not so evident, reflections of the superstructure do not appear in the X-ray diffraction pattern, and the structure can be described by the space group *Fd*-3 m.

According to Delsante et al.^11^, formation of the regular C15 structure (space group *Fd*-3 m) is expected for the RNi_2_ compounds with the enthalpy of formation Δ_f_*H*^o^ at 300 K of less than − 40 kJ/mol. The enthalpies of formation Δ_f_*H*^o^ of HoNi_2_ and ErNi_2_ equal to –48 and –50 kJ/mol, respectively, suggest the emergence of the regular C15 structure in these compounds, which was indeed confirmed in our earlier work ^20^. However, in the case of Ho_0.5_Er_0.5_Ni_2_ and Ho_0.25_Er_0.75_Ni_2_ solid solutions, this rule is not confirmed. Additional vacancies are induced and are responsible for the formation of the superstructure. Vacancies arise as structural defects resulting from differences in the atomic radii of elements comprising a solid solution. Owing to the difference in the atomic radii, Ho–Ni and Er–Ni bonds in the solid solutions differ in length; this fact has a direct impact on the formation of vacancies. Similar results were obtained for the Ho distribution in Tb_1-x_Ho_x_Ni_2_ solid solutions ^22^ and are in line with the data obtained for the other ternary Laves-phase solid solutions, e.g., Tb_1-x_Dy_x_Ni_2_^23^ studied previously.

According to the data given in Fig. 1, small amounts of Ho_2_O_3_ and Er_2_O_3_ impurity phases are present in the Ho_0.5_Er_0.5_Ni_2_ and Ho_0.25_Er_0.75_Ni_2_ samples, the total content of which is not more than 3 wt. %. For Ho_0.75_Er_0.25_Ni_2_, the lattice parameter is equal to 7.1462 Å. For the two consecutive substitutions, the lattice parameter decreases as the Er content increases to *x* = 0.75. This is due to the fact that, in accordance with the lanthanide contraction, the radius of Er atoms (176 pm) is smaller than that of Ho (177 pm). It should be noted that the parent compounds, similarly to the Ho_0.75_Er_0.25_Ni_2_ compound, solidify with the formation of the cubic C15 crystal structure.

**Figure 2:**
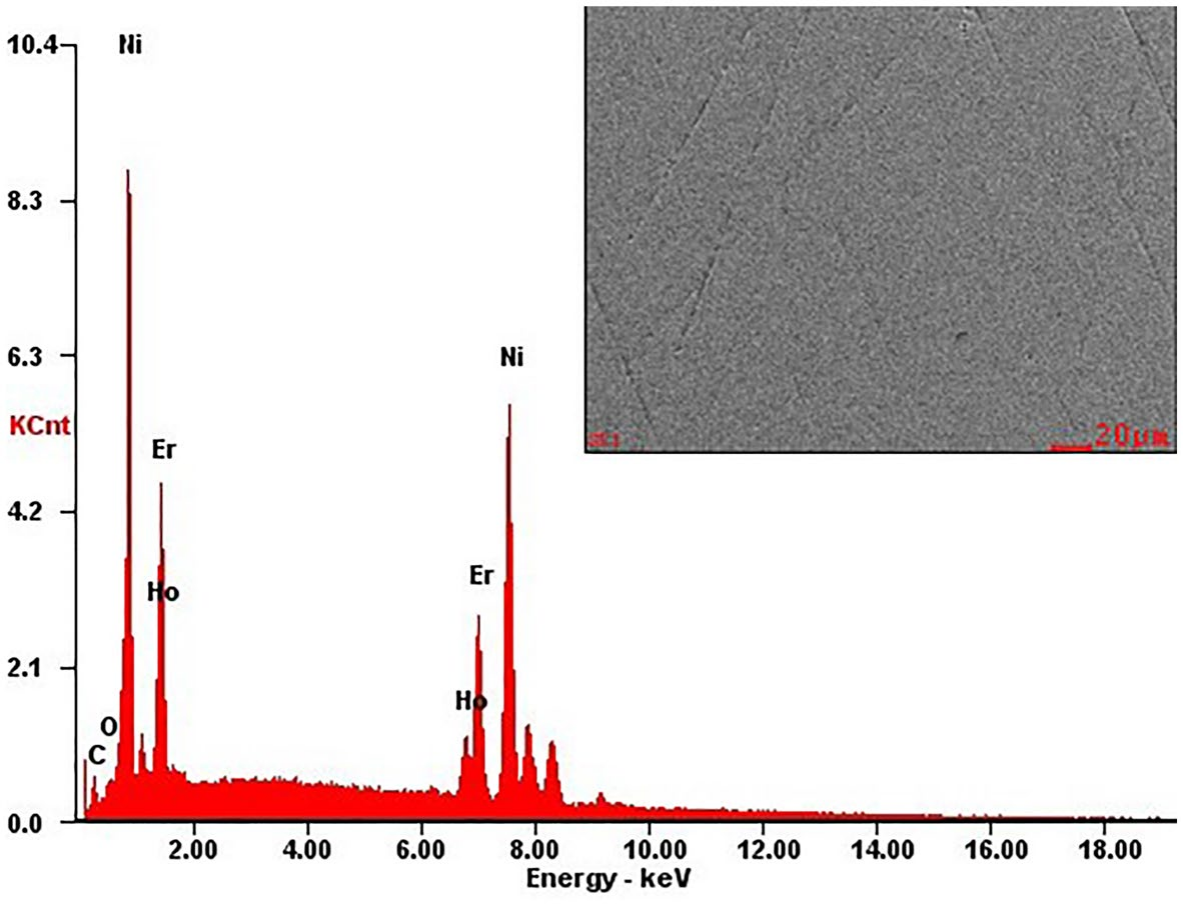
Energy-dispersive x-ray (EDX) analysis data for the Ho_0.25_Er_0.75_Ni_2_ solid solution and SEM image (with secondary electrons contrast) of the typical polished surface.

The typical SEM image and EDX studies of the characteristic microstructure of the polished section as representative of Ho_0.25_Er_0.75_Ni_2_ are shown in Fig. 2. The EDX analysis performed for large areas of Ho_0.25_Er_0.75_Ni_2_ sample showed that its chemical composition is consistent with the nominal one (the Ho, Er, and Ni contents are 8.07, 26.13, and 65.81 at.%, respectively). Similar results were also obtained for the other samples.

### Evaluation of magnetocaloric effect by indirect method

In general, the heat capacity of metallic magnetic systems can be considered as the sum of the independent electron, lattice (phonon) and magnetic contributions:

1 $$C_{{{\text{tot}}}} \left( T \right) = C_{{{\text{el}} + {\text{ph}}}} \left( T \right) + C_{{{\text{mag}}}} \left( T \right).$$

The electron and phonon contributions to the heat capacity can be calculated by the formula:

2 $$C_{{{\text{el}} + {\text{ph}}}} \left( T \right) = \gamma T + 9NR\left( {\frac{T}{{\Theta_{D} }}} \right)^{3} \mathop \int \limits_{0}^{{\Theta_{D} /T}} \frac{{x^{4} e^{x} }}{{\left( {e^{x} - 1} \right)^{2} }}dx,$$

where the first term represents an electron heat capacity and the second term corresponds to a phonon contribution in accordance with Debye’s model; γ is the Sommerfeld coefficient; Ɵ_D_ is the Debye temperature; *N* = 3 is the number of atoms per formula unit; and R is the molar gas constant.

To isolate the electron–phonon contribution from the total heat capacity of measured Ho_1-x_Er_x_Ni_2_ solid solutions, the curves of the measured heat capacity for an isostructural non-magnetic compound, LaNi_2_ were used. It was found that, in the low-temperature range 1.8–4 K, the linear dependence of the *C*_P_/*T* vs *T*^2^ in LaNi_2_ can be fitted with the Sommerfeld coefficient γ = 6.6 mJ/molK and the Debye temperature Ɵ_D_ = 242 K^24^. However, we have found that the best fittings for the wide temperature range 2–100 K, for all the studied samples, could be obtained by fixing the parameter γ = 3.8 mJ/molK^2^, while the Debye temperature Ɵ_D_ of the Ho_1−x_Er_x_Ni_2_ system, similarly to that of the Dy_1−x_Er_x_Ni_2_ system^25^, increases as the Er content increases from 254 K for x = 0.25 to 271 K for x = 0.75. It should be noted that the Debye temperature values obtained are comparable with those of other known RNi_2_ compounds. By comparison, the Ɵ_D_ values for TbNi_2_, DyNi_2_ and ErNi_2_ compounds were reported to be 261, 250 and 264 K, respectively^26–28^. Table 1 shows the Debye temperatures and the γ values calculated by this method.

**Table 1:** Curie temperature *T*_C_, low-temperature limit of Debye temperature, Sommerfeld coefficient γ and maximum magnetic entropy determined theoretically (calculated) and experimentally at a temperature of 100 K for the investigated Ho_1-x_Er_x_Ni_2_ intermetallic compounds determined from the heat capacity measurements.

Compound	*T*_C_ (K)	Ɵ_D_ (K)	γ (mJ/molK^2^)	*S*_mag_ (J/molK)	
				theor., *R*ln(2* J* + 1)	*T* = 100 K
Ho_0.75_Er_0.25_Ni_2_	12.0	254	3.8	23.43	21.4
Ho_0.5_Er_0.5_Ni_2_	9.7	260	3.8	23.30	21.4
Ho_0.25_Er_0.75_Ni_2_	7.7	271	3.8	23.18	22.1

The characteristic temperatures were determined to an accuracy of ± 0.1 K. The magnetic entropy was calculated to an accuracy of ± 0.1 J/molK.

**Figure 3:**
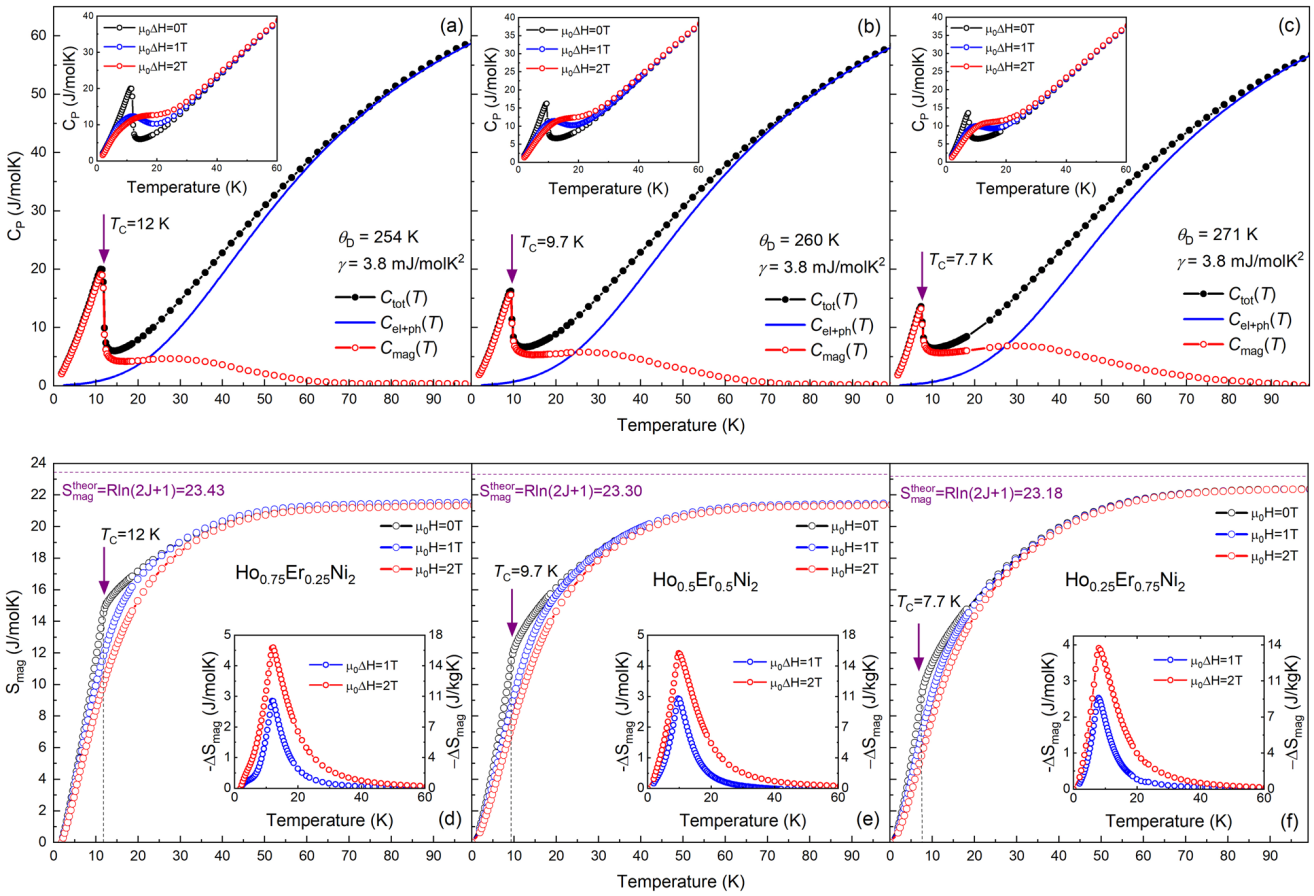
Total heat capacity C_tot_(T) of Ho_0.75_Er_0.25_Ni_2_ (**a**), Ho_0.5_Er_0.5_Ni_2_ (**b**) and Ho_0.25_Er_0.75_Ni_2_ (**c**) measured in zero magnetic field. The calculated sum of electronic and phonon contributions *C*_el+ph_ as well as estimated magnetic contribution *C*_mag_. The insets of (**a**)–(**c**) show the heat capacity as a function of temperature measured in zero, 1- and 2-T magnetic fields, respectively. Temperature dependences of the magnetic entropy *S*_mag_(*T*) for Ho_0.75_Er_0.25_Ni_2_ (**d**), Ho_0.5_Er_0.5_Ni_2_ (**e**) and Ho_0.25_Er_0.75_Ni_2_ (**f**) in zero, 1- and 2-T magnetic fields. The horizontal dotted lines correspond to the theoretical maximum value *S*_mag_ = *R*ln(2* J* + 1) and the vertical dotted lines correspond to the magnetic phase transition temperature *T*_C_. Insets show the magnetic entropy change Δ*S*_mag_ measured for magnetic field changes of 1 and 2 T.

Figures 3a–c show the temperature dependences of the total heat capacity, *C*_tot_(*T*), of the Ho_1-x_Er_x_Ni_2_ solid solutions in zero magnetic field. Filled symbols correspond to the experimental data, and open symbols correspond to the magnetic part of heat capacity, *C*_mag_(*T*), obtained after subtraction of the electron and phonon contribution, *C*_el+ph_(*T*), which was estimated by Debye function (solid lines in Fig. 3a-c) according to the Eq. (2).

In the absence of magnetic field, the temperature dependence of the heat capacity shows a peak corresponding to magnetic phase transition typical of ferromagnetic compounds. The Curie temperatures *T*_C_ of the Ho_0.75_Er_0.25_Ni_2_ (Fig. 3a), Ho_0.5_Er_0.5_Ni_2_ (Fig. 3b), and Ho_0.25_Er_0.75_Ni_2_ (Fig. 3c) compounds are 12.0, 9.7, and 7.7 K, respectively.

Insets in Fig. 3a–c show the heat capacity, as a function of temperature, measured in zero, 1- and 2-T magnetic fields. The feature observed for all of the studied compositions is the broadening of the *C*_tot_(*T*) peak and reduction of its height, which takes place with the increasing applied magnetic field.

The magnetic part of the entropy *S*_mag_(*T*) was calculated by integrating the dependence *C*_mag_(*T*)/*T* for each composition (Fig. 3d–f). This procedure is valid when assuming that the electronic and lattice contributions are field-independent and in the case of an adiabatic field change process, when Δ*S*_tot_ = 0^29^. The fact that the dependence of entropy exhibits a strong tendency to saturation, but the entropy does not approach the theoretical maximum value *S*_mag_ = *R*ln (2* J* + 1) (where *J* is the total angular momentum of a rare earth ion) at the Curie temperatures can be explained by peculiarities in the ground-state level splitting by the crystal electric field (CEF) when several CEF levels are separated from others by a substantial energy gap^30^. Similar behavior was observed for other pseudo-binary Laves-phase compounds^25,31,32^. According to the theoretical calculations, the maximum magnetic entropy should equal to 23.2–23.4 J/molK. In the case of the tested solid solutions, the maximum value of *S*_mag_ for Ho_0.75_Er_0.25_Ni_2_ and Ho_0.5_Er_0.5_Ni_2_ is 21.4 J/molK at 100 K and is 22.3 J/molK for Ho_0.25_Er_0.75_Ni_2_. This means that almost the total magnetic entropy associated with the magnetic process is utilized.

The temperature behaviour of the magnetic entropy in 1- and 2-T magnetic fields shows that the applied magnetic field leads to the decrease in *S*_mag_ near *T*_C_. In particular, the maximum value of *S*_mag_ for Ho_0.75_Er_0.25_Ni_2_ near *T*_C_ decreases from 15.1 to 10.5 J/molK in the applied magnetic field. The temperature dependences of the isothermal magnetic entropy change Δ*S*_mag_(*T*) calculated using the heat capacity data according to the procedure reported in^25^ and caused by 1- and 2-T magnetic field change, are shown in insets in Fig. 3d–f. For a magnetic field change of 0–2 T, the experimental maximum − Δ*S*_mag_ in the case of the Ho_0.75_Er_0.25_Ni_2_ compound reaches the highest value of 4.6 J/mol K (16.3 J/kg K) near 12.1 K and, as the Er content increases, becomes lower and equals to 3.9 J/mol K (13.7 J/kg K) for the Ho_0.25_Er_0.75_Ni_2_ sample near 8 K.

**Figure 4:**
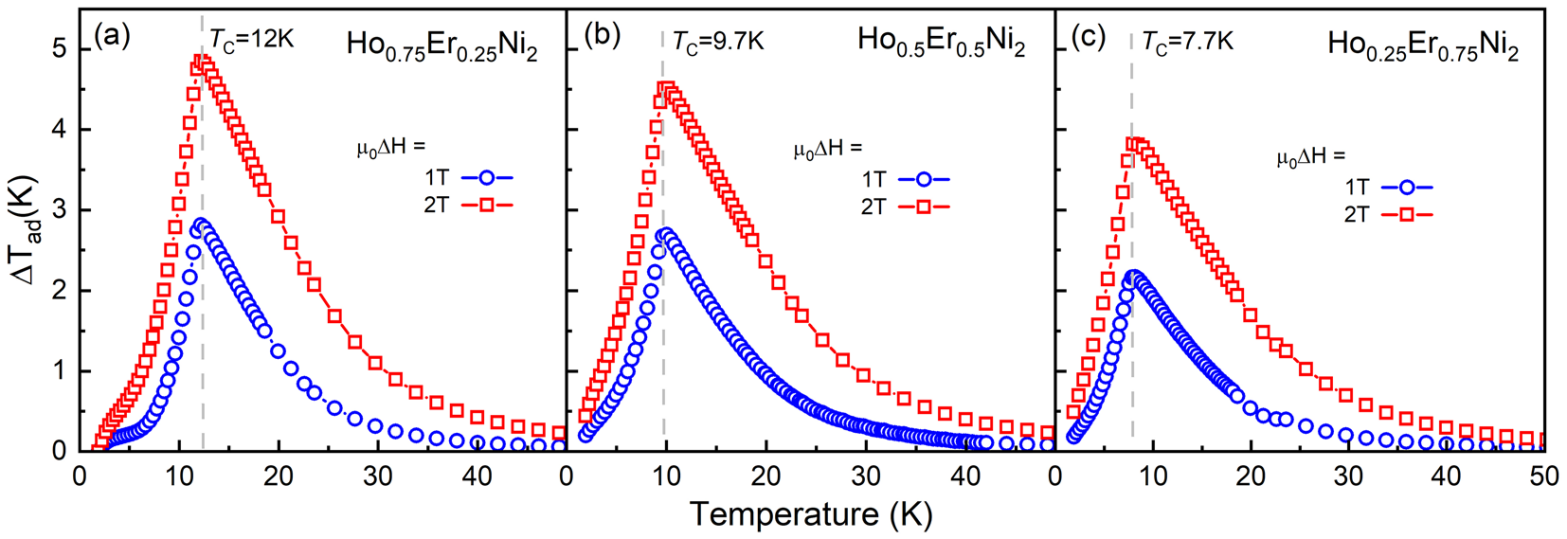
Temperature dependences of the adiabatic temperature change, Δ*T*_ad_, of Ho_0.75_Er_0.25_Ni_2_ (**a**), Ho_0.5_Er_0.5_Ni_2_ (**b**) and Ho_0.25_Er_0.75_Ni_2_ (**c**) calculated from heat capacity data measured in 1- and 2-T magnetic fields.

Figure 4a–c show dependences of the adiabatic temperature change, Δ*T*_ad_, for Ho_1−x_Er_x_Ni_2_ with x = 0.25, 0.5, and 0.75, which were derived from the heat capacity data obtained in 1- and 2-T magnetic fields. As is seen, the increase in the applied magnetic field leads to an increase in the adiabatic temperature change near *T*_C_. Both at 1- and 2-T magnetic field changes, the highest magnetocaloric effect was observed for Ho_0*.*75_Er_0*.*25_Ni_2_. The maximum Δ*T*_ad_ for Ho_0*.*75_Er_0*.*25_Ni_2_ reaches 2.8 K (4.9 K) at 12.0 K, and, with increasing Er content, the maximum peak value of Δ*T*_ad_ decreases to 2.2 K (3.9 K) for Ho_0*.*25_Er_0*.*75_Ni_2_ at 7.7 K for a magnetic field change of 1 (2) T. Table 2 summarizes the data on the experimental isothermal magnetic entropy change Δ*S*_mag_(*T*) and adiabatic temperature change Δ*T*_ad_(*T*) for low external magnetic field changes, which were estimated by the indirect method using the heat capacity data.

**Table 2:** Magnetocaloric properties for the selected binary RNi_2_ compounds and for the investigated Ho_1-x_Er_x_Ni_2_ solid solutions estimated from heat capacity measurements for magnetic field changes of 1 and 2 T. *T*_C_ is the magnetic phase transition temperature; -Δ*S*_mag_ is the maximum magnetic entropy change; Δ*T*_ad_ is the maximum adiabatic temperature change; *RC* is the refrigerant capacity; *RCP* is the relative cooling power; and *TEC* is the temperature averaged entropy change.

Compound	*T*_C_ (K)	−Δ*S*_mag_ (J/kgK)		Δ*T*_ad_ (K)		*RC/RCP* (J/kg)		*TEC*(3)/ *TEC*(10) (J/kgK)	
		0–1 T	0–2 T	0–1 T	0–2 T	0–1 T	0–2 T	0–1 T	0–2 T
TbNi_2_^37^	37.1	3.4	6.5	1.4	2.4	34/44	94/122	3.2/2.9	6.4/6.0
DyNi_2_^37^	21.8	6.3	11.1	2.4	3.8	48/63	117/154	5.9/4.8	11.1/9.1
HoNi_2_^20^	13.5	8.8	14.6	2.7	4.6	55/72	128/171	8.0/6.2	14.9/11.4
Ho_0.75_Er_0.25_Ni_2_	12.0	10.0	16.2	2.8	4.9	49/65	117/155	8.9/4.9	15.1/9.1
Ho_0.5_Er_0.5_Ni_2_	9.7	10.3	15.5	2.7	4.5	52/72	123/163	9.0/6.8	15.1/11.8
Ho_0.25_Er_0.75_Ni_2_	7.7	8.8	13.7	2.2	3.9	45/58	102/133	8.0/5.6	12.6/10.4
ErNi_2_^20^	6.2	8.6	13.0	2.2	3.8	43/55	96/122	7.8/5.3	12.1/9.3
Composite 1	–	6.7	–	–	–	57/67	–	6.6/5.6	–
Composite 2	–	–	12.0	–	–	–	121/150	–	11.9/10.4

The values of the characteristic temperatures were calculated to an accuracy of ± 0.1 K. The magnetic entropy was calculated to an accuracy of ± 0.1 J/kgK.

To compare the refrigeration properties of Ho_1−x_Er_x_Ni_2_ with those of the other previously investigated RNi_2_ compounds, the refrigerant capacities (*RC*), relative cooling power (*RCP*) and temperature averaged entropy change (*TEC*) were estimated. The first parameter is a measure of the amount of heat that can be transferred between the cold and hot sinks in one ideal refrigeration cycle and was estimated by integrating the Δ*S*_mag_(*T*) curve over the full width at half maximum^33,34^. It should be noted that, as the magnetic entropy change decreases owing to the Er doping in Ho_1−x_Er_x_Ni_2_, the *RC* also reduces, but it is still high, namely, ~ 45 J/kg and ~ 102 J/kg for a field change of 1 and 2 T, respectively.

The second parameter is defined as ǀΔ*S*magǀ^(max)^ × δ*T*_FWHM_, where δ*T*_FWHM_ denotes the full width temperature span of ǀΔ*S*magǀ vs. *T* curve at its half maximum^35^. As the Er content increases, the *RCP* values decrease from 65 J/kg for Ho_0.75_Er_0.25_Ni_2_ to 58 J/kg for Ho_0.25_Er_0.75_Ni_2_ at the 1-T magnetic field change and from 155 J/kg for Ho_0.75_Er_0.25_Ni_2_ to 133 J/kg for Ho_0.25_Er_0.75_Ni_2_ at the 2-T magnetic field change. It should be noted that, in the case of the Ho_0.5_Er_0.5_Ni_2_ solid solution, there are slight deviations for both the obtained *RC* and *RCP* values from the expected ones.

The third parameter, the temperature averaged entropy change (*TEC*), was introduced by Griffith et al.^10^ and the magnitude is calculated by the following formula:

3 $$TEC\left( {\Delta T_{{{\text{lift}}}} } \right) = \frac{1}{{\Delta T_{{{\text{lift}}}} }}\mathop {\max }\limits_{{T_{{{\text{mid}}}} }} \left\{ {\mathop \int \limits_{{T_{{{\text{mid}}}} - \frac{{\Delta T_{{{\text{lift}}}} }}{2}}}^{{T_{{{\text{mid}}}} + \frac{{\Delta T_{{{\text{lift}}}} }}{2}}} \left| {\Delta S_{{\text{M}}} } \right|\left( T \right)_{{\mu_{0} \Delta H,T}} dT} \right\}$$

where Δ*T*_lift_ is the desired lift of temperature and *T*_mid_ is the temperature of the center of the *TEC* and is determined by maximizing the *TEC* value. Accordingly, two different ∆*T*_lift_ values of 3 and 10 K are chosen to calculate *TEC* for the Ho_1−x_Er_x_Ni_2_ solid solutions under study. The resulted values of *TEC* (3 K) and *TEC* (10 K) at µ_0_∆*H* = 1 T oscillate between 8.0–9.4 and 4.9–6.8 J/kgK and, at µ_0_∆*H* = 2 T, oscillate between 12.6–15.1 and 9.1–11.8 J/kgK, respectively.

The obtained values are of a high level and are comparable to those obtained for other promising low temperature magnetocaloric materials, such as TbNi_2_^36,37^, DyNi_2_^37,38^, ErNi_2_, HoNi_2_^20^, Dy_1−x_Er_x_Ni_2_^25^, Tb_1−x_Ho_x_Ni_2_^22^, TmCoAl^39^, ErRu_2_Si_2_^40^, or HoNi_2_B_2_C^41^.

Due to the fact that the ideal Ericsson cycle employs a constant value of Δ*S*_mag_ in the temperature range of refrigeration, which is necessary for improving regeneration processes, composite materials were considered. It is expected that a composite material formed by at least two magnetic Ho_1-x_Er_x_Ni_2_ compounds differing in the Er concentration could exhibit a “table-like” behavior of MCE in a wider temperature range. In this context, according to a procedure proposed in Refs.^20,42,43^, numerical simulations were done to construct a composite material formed by Ho_1−x_Er_x_Ni_2_ compounds. The isothermal magnetic entropy change of a magnetic composite ǀΔ*S*_mag_ǀ^comp^ based on *N* kinds of magnetic materials is equal to the sum of their magnetic entropy changes ǀΔ*S*_mag_ǀ_j_ weighted by a molar ratio *y*_j_. In our case, for a magnetic field change of 0–1 T (composite 1), optimal molar ratios are y_1_ = 0.599 for Ho_0.25_Er_0.75_Ni_2_, y_2_ = 0.046 for Ho_0.5_Er_0.5_Ni_2_, and y_3_ = 0.355 for Ho_0.75_Er_0.25_Ni_2_, while, in the case of a magnetic field change of 0–2 T (composite 2), two compounds are sufficient with y_1_ = 0.706 for Ho_0.25_Er_0.75_Ni_2_ and y_2_ = 0.294 for Ho_0.75_Er_0.25_Ni_2_.

**Figure 5:**
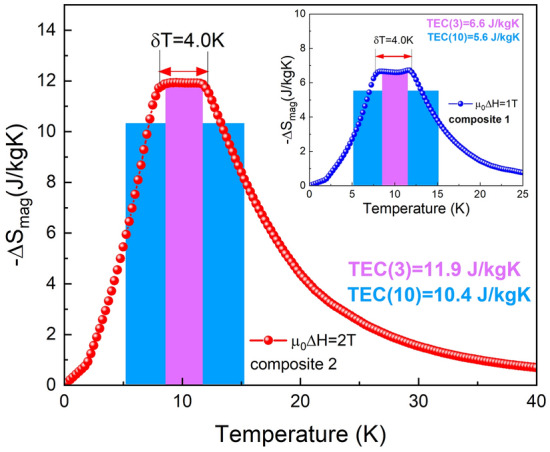
Temperature dependences of the isothermal magnetic entropy change -Δ*S*_mag_(*T*) and temperature averaged entropy change *TEC*(3) and *TEC*(10) calculated for composites based on the investigated compounds, for magnetic field changes of 1 T (inset) and 2 T.

Figure 5 shows the calculated isothermal magnetic entropy changes for the composite based on Ho_1−x_Er_x_Ni_2_ compounds, which are obtained for magnetic field changes of 1 and 2 T. It should be noted that, both in 1- and 2-T magnetic field changes, the maximum magnetic entropy change of the composite material exhibits an almost constant value of ǀΔ*S*_mag_ǀ^comp^ that is around 6.7 J/kgK for µ_0_Δ*H* = 1 T and 12 J/kgK for µ_0_Δ*H* = 2 T. For both composites, calculated ǀΔ*S*_mag_ǀ^comp^ remains almost unchanged in a temperature range of 8 to 12 K. These results suggest that, in order to design the appropriate composition of a refrigerant, it is necessary to evaluate the corresponding optimal molar ratios using the value of external magnetic field change at which the refrigerator should operate. To compare the magnetocaloric performance of the proposed composites with that of their constituents, the values of *RC, RCP,* and *TEC* have been calculated. The magnitudes computed by the methods described earlier for both composites are of a high level and are comparable to those of the individual solid-solution constituents; the value of *RC*(*RCP*) for composite 1 (µ_0_Δ*H* = 1 T) is equal to 57(67) J/kg and, for composite 2 (µ_0_Δ*H* = 2 T), it is 122(150) J/kg. The *TEC*(3) values obtained for both composites are comparable to their maximum isothermal magnetic entropy change values, which result directly from the scope of ∆*T*_lift_ values. In the case of *TEC*(10), the values are slightly smaller in comparison with *TEC*(3); however, they are still of a high level and comparable to those of the solid solution constituents (see Table 2).

### Evaluation of the magnetocaloric effect with direct measurements

**Figure 6:**
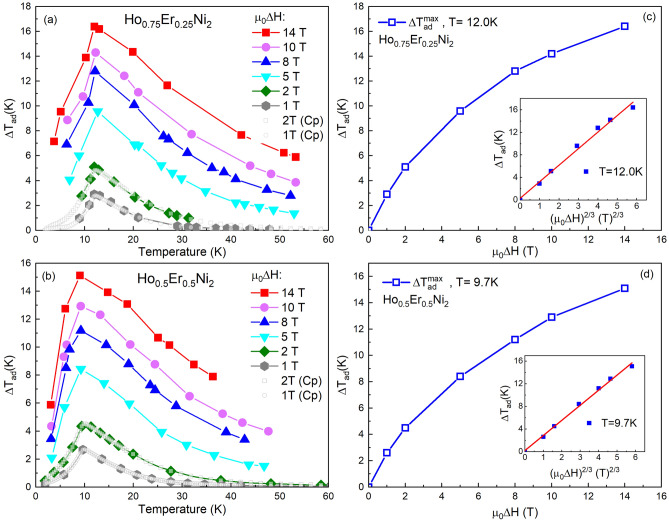
Temperature dependences of the adiabatic temperature change, Δ*T*_ad_, as obtained from the heat capacity data (filled symbols) and from direct measurements (open symbols) for Ho_0.75_Er_0.25_Ni_2_ (**a**) and Ho_0.5_Er_0.5_Ni_2_ (**b**) at different magnetic field changes µ_0_Δ*H* and maximum adiabatic temperature change, Δ*T*_ad_^max^, for Ho_0.75_Er_0.25_Ni_2_ (**c**), Ho_0.5_Er_0.5_Ni_2_ (**d**) as a function of the magnetic field change, µ_0_Δ*H*. Insets show the Δ*T*_ad_ as a function of (µ_0_Δ*H*)^2/3^. Solid lines present the relation Δ*T*_ad_ = *A*(µ_0_Δ*H*)^2/3^, with *A* listed in Table 3.

The adiabatic temperature change Δ*T*_ad_ caused by the magnetic field change µ_0_Δ*H*, i.e., the magnetocaloric effect, has been additionally determined by direct temperature measurements in the range of magnetic fields up to 14 T. Figures 6a,b show experimental Δ*T*_ad_ vs. the initial temperature, as obtained in the magnetizing process and for comparison, derived from heat capacity data, for Ho_0.75_Er_0.25_Ni_2_ and Ho_0.5_Er_0.5_Ni_2_, respectively. The initial field was zero in all cases. Note, that the results are very similar for both methods. As expected, the increase of the applied magnetic field leads to an increase in Δ*T*_ad_. The maximum value of Δ*T*_ad_ at µ_0_Δ*H* = 14 T reaches 16.4 K at *T*_C_ for Ho_0.75_Er_0.25_Ni_2_, and 15.1 K at *T*_C_ for Ho_0.5_Er_0.5_Ni_2_. The maxima of Δ*T*_ad_ obtained at 1- and 2-T magnetic field changes by both direct and indirect methods have been detected at the same temperature and the determined values are in good agreement.

Directly measured maximum Δ*T*_ad_ as a function of the final magnetic field is plotted in Fig. 6c,d. For both Ho_0.75_Er_0.25_Ni_2_ and Ho_0.5_Er_0.5_Ni_2_ solid solutions, Δ*T*_ad_ grows nonlinearly with increasing µ_0_Δ*H*. Characteristic quantity Δ*T*_ad_/µ_0_Δ*H* decreases from 2.8 K/T at 1 T to 1.2 K/T at 14 T for Ho_0.75_Er_0.25_Ni_2_, and from 2.7 K/T at 1 T to 1.1 K/T at 14 T for Ho_0.5_Er_0.5_Ni_2_.

Experimental results can be interpreted within the framework of the thermodynamic Landau theory. According to this theory, the equation for the magnetization of paraprocess near the Curie temperature can be written as^44^

4 $$\alpha \cdot M + \beta \cdot M^{3} = H,$$

were *α* and *β* are the thermodynamic Landau coefficients and *M* is magnetization. The expression for MCE caused by an adiabatic change of magnetization is

5 $$dT = - \frac{T}{{C_{M,P} }}\left( {\frac{\partial H}{{\partial T}}} \right)_{M} dM.$$

Near the Curie temperature, the *β* coefficient is only weakly dependent on temperature and therefore the temperature derivative from Eq. (4) equals to

6 $$\left( {\frac{\partial H}{{\partial T}}} \right)_{M} = \alpha_{1} M.$$

Substituting Eq. (6) into Eq. (5) we obtain

7 $$dT = - \frac{{\alpha_{1} T}}{{C_{M,P} }}MdM.$$

Integration of the expression(7) leads to

8 $$\Delta T = \mathop \int \limits_{0}^{I} \frac{{\alpha_{1} T}}{{_{M,P} }}MdM = \frac{{\alpha_{1} T}}{{2_{M,P} }}M^{2} .$$

Thus, MCE must obey the law of proportionality to the squared magnetization in the region of paraprocess ^44^

9 $$\Delta T = k \cdot M^{2}$$

where $k = \frac{{\alpha_{1} T}}{{2_{M,P} }}$. This was confirmed experimentally by Weiss and Piccard ^45^. The magnetic field dependence of Δ*T* can be described by the equation of state following from the thermodynamic Landau theory

10 $$\frac{\alpha + \gamma P}{{k^{1/2} }} + \frac{\beta }{{k^{3/2} }}\Delta T = \frac{H}{{\Delta T^{1/2} }}.$$

As is seen from Eq. (10), Δ*T* ~ *H*/Δ*T*^1/2^ or Δ*T* ~ *H*^2/3^. To check the applicability of the thermodynamic Landau theory for the description of our experimental results, the adiabatic temperature change Δ*T*_ad_ was plotted as a function of (µ_0_Δ*H*)^2/3^, as is shown in insets in Fig. 6c,d. The linear behavior of the dependences for both investigated compounds near their Curie temperature demonstrates a good agreement between the experimental results and thermodynamic Landau theory.

By plotting the maximum Δ*T*_ad_ value versus (µ_0_Δ*H*)^2/3^ and using an equation: Δ*T*_ad_ = *A*(µ_0_Δ*H*)^2/3^, where *A* is a characteristic parameter of magnetocaloric materials, one can obtain information about the magnetocaloric properties of investigated samples^46^. By fitting the experimental data, we find *A* = 2.9 K/T^2/3^ for Ho_0.75_Er_0.25_Ni_2_ and *A* = 2.6 K/T^2/3^ for Ho_0.5_Er_0.5_Ni_2_. These values are comparable with those obtained for the parent compounds and other binary Laves-phase compounds and are also comparable with the values of the most efficient magnetic refrigerants, such as Gd (*A* = 3.83 K/T^2/3^) and LaFe_11.2_Si_1.8_ (*A* = 2.16 K/T^2/3^)^46^. The data obtained by direct measurements are gathered in Table 3.

**Table 3:** Experimental data characterizing the adiabatic temperature change, Δ*T*_ad_, due to MCE caused by the magnetic field change, µ_0_Δ*H*, for the selected binary RNi_2_ intermetallic compounds and for Ho_0.75_Er_0.25_Ni_2_ and Ho_0.5_Er_0.5_Ni_2_ solid solutions. *A* is the coefficient from equation Δ*T*_ad_ = *A*(µ_0_Δ*H*)^2/3^. The data were obtained by direct measurements of Δ*T*_ad_ during the field change, µ_0_Δ*H*, achieved by using the extraction method in a Bitter magnet. The values of Δ*T*_ad_ marked with ^**‡**^ symbol are estimated by the extrapolation of the Δ*T*_ad_ = *A*(µ_0_Δ*H*)^2/3^ relation.

Compound	*T*_C_ (K)	Δ*T*_ad_ (K)						*A* (K/T^2/3^)
		0–1 T	0–2 T	0–5 T	0–8 T	0–10 T	0–14 T	
TbNi_2_^37^	37.1	1.5^**‡**^	2.4	4.6	6.2	6.9	8.4^**‡**^	1.45
DyNi_2_^37^	21.8	2.3	3.6	7.1	9.2	10.6	13.4^**‡**^	2.31
HoNi_2_^20^	13.5	2.8^**‡**^	4.2	8.7	11.4	12.9	16.3^**‡**^	2.8
Ho_0.75_Er_0.25_Ni_2_	12.0	2.9	5.1	9.6	12.7	13.9	16.3	2.9
Ho_0.5_Er_0.5_Ni_2_	9.7	2.6	4.5	8.3	11.2	12.9	15.1	2.6
ErNi_2_^20^	6.2	2.1^**‡**^	3.5	6.2	8.8	9.8	12.2^**‡**^	2.1

The characteristic temperatures were calculated to an accuracy of ± 0.1 K.

## Discussion

The present study, by an example of Ho_1−x_Er_x_Ni_2_, develops the concept of magnetocaloric efficiency of the rare-earth Laves-phase solutions starting from their magneto thermodynamic properties and then proceeds illustrating their potentiality as components of magnetocaloric composites.

The analysis of the structural data obtained for the Ho_1−x_Er_x_Ni_2_ solid solutions confirms the similarity of the structures of the parent HoNi_2_ and ErNi_2_ binary compounds and the Ho diluted compound with x = 0.25, which have the regular C15 cubic structure (Laves phase). The subsequent introduction of Ho to x = 0.5 and 0.75 leads to the formation of a cubic superstructure that is due to a regular arrangement of vacancies at rare earth sites and decreases the crystal lattice symmetry (space group *F*-43* m*). The superstructure is characterized by the doubled lattice parameter.

The measurements of the heat capacity were performed for the compounds, the phase and chemical compositions of which were well characterized. The appearance of Er atoms in the rare-earth sublattice results in the common magnetic dilution consisted in weakening the exchange interactions, which is accompanied by the decrease in the ordering temperature of the Ho_1−x_Er_x_Ni_2_ system. Thus, its linear variations, namely, decrease in the Curie temperature of the system from 12.0 K (for Ho_0.75_Er_0.25_Ni_2_) to 7.7 K (for Ho_0.25_Er_0.75_Ni_2_) are realized by the mutual substitutions of rare-earth components.

The magnetothermodynamics properties of the three-component solid solutions were characterized by indirect evaluation and direct measurements of magnetocaloric potentials in a wide range of magnetic fields. The possibility of precise tailoring the magnetocaloric potentials to a certain temperature range was demonstrated. As the Er content increases, the maximum magnetic entropy change decreases from 16.2 J/kgK for Ho_0.75_Er_0.25_Ni_2_ at 12 K and reaches 13.7 J/kgK at 7.7 K for Ho_0.25_Er_0.75_Ni_2_ for a magnetic field change of 2 T. The maximum adiabatic temperature change Δ*T*_ad_ for Ho_0.75_Er_0.25_Ni_2_ in the 2-T magnetic field change is equal to 4.9 K at 12 K, and with increasing Er content, the Δ*T*_ad_ value decreases to 3.9 K for Ho_0.25_Er_0.75_Ni_2_ in the vicinity of *T*_C_. The maximum values of the adiabatic temperature change, determined by the direct measurements, reach 16.4 K near 12.0 K for Ho_0.75_Er_0.25_Ni_2_ and 15.1 K near 9.7 K for Ho_0.5_Er_0.5_Ni_2_ at µ_0_Δ*H* = 14 T. The maximum values of Δ*T*_ad_, obtained at 1- and 2-magnetic fields, obtained by direct and indirect methods are in good agreement. The directly measured adiabatic temperature changes near the Curie temperature in high magnetic fields were compared with the values obtained based on the Landau theory for the second-order phase transitions. It was demonstrated that, the magnetic field dependence of Δ*T*_ad_ obeys the (µ_0_Δ*H*)^2/3^ function with the parameter *A*, which characterizes intrinsic properties of refrigerants, and the Landau theory of second-order phase transitions is applicable for Δ*T*_ad_ description in high magnetic fields.

Additionally, the availability of the magnetocaloric potentials experimentally estimated for the individual three-component Ho_1−x_Er_x_Ni_2_ solid solutions allows us to simulate optimal molar ratios to construct the composites to be considered as a refrigerant material in magnetic refrigerators performing an Ericsson cycle at low temperatures. These theoretical results based on the real empirical data are very optimistic and are of interest to perform further experimental studies. The results of the simulation indicate that the proposed composite exhibits a high potential for the application in magnetic refrigeration devices, especially in the cryogenic temperature range.

## Methods

### Sample preparation, structural analysis, heat capacity, and direct magnetocaloric measurements

Ho_1-x_Er_x_Ni_2_ alloys with x = 0.25, 0.5, and 0.75 were prepared by repeated arc-melting of appropriate amounts of starting metals in a high-purity argon atmosphere at a pressure of 1.5 atm; starting metallic components of at least 99.9 wt.% (rare-earths) and 99.99 wt.% (Ni) purity were used. The obtained ingots were wrapped separately with Mo foil and subsequently subjected to homogenizing annealing in an argon-filled quartz tube. The annealing process was performed at 1123 K for one month; subsequently, the ingots were subjected to slow furnace cooling to room temperature to ensure their uniform cooling, exclude the fixation of high-temperature structural state of compounds, and to obtain their equilibrium state. The elemental composition was assessed by energy dispersive X-ray (EDX) spectroscopy with the simultaneous study of the sample’s microstructure by scanning electron microscopy (SEM) using an FEI Nova Nano SEM 230 scanning electron microscope (operating at an accelerating voltage of 20 kV) equipped with an energy-dispersive spectrometer (EDAX Genesis XM4). The crystal structure was determined by X-ray diffraction (XRD) analysis, which was carried out at room temperature using powdered samples and an Ultima IV Rigaku (Japan) diffractometer equipped with a “D/teX” high-speed semiconductor detector. X-ray diffraction patterns were taken in an angular range of 9–100^0^ at a step of 0.02^0^ using CuKα radiation and a fluorescent correction regime.

Heat capacity was measured in the 2–100 K temperature range in zero, 1- and 2-T magnetic fields by a relaxation method using a PPMS-14 installation (Quantum Design, USA). The direct measurements of Δ*T*_ad_ were performed in the 4.2–50 K temperature range in magnetic fields up to 14 T using the original setup, which is based on the extraction method and allows us to perform direct measurements of the adiabatic temperature change ^47^. Steady magnetic fields up to 14 T were generated by a Bitter-type magnet, and a maximum field-change rate of ~ 6 T/s was obtained by moving the sample in and out of the applied magnetic field.
